# Foreign and Regional Languages Make You Less Deontological

**DOI:** 10.5334/joc.346

**Published:** 2024-01-29

**Authors:** Francesca Peressotti, Greta Pianezzola, Marta Battistutta, Michele Miozzo

**Affiliations:** 1 Dipartimento di Psicologia dello Sviluppo e della Socializzazione, University of Padua, Italy; 2 Padua Neuroscience Center, University of Padua, Italy; 3 Department of Psychology, Columbia University, New York, US

**Keywords:** bilingualism, moral decisions, process dissociation procedure, foreign language effect

## Abstract

Previous studies have shown that foreign languages can change people’s responses to moral dilemmas, making them more likely to choose harm (e.g., to kill one individual in order to save a few lives). Regional languages have also been shown to make sacrificial choices more likely. Regional languages are typically acquired early and used routinely among family and acquaintances, thus differing from foreign languages that are typically acquired later and used rather sporadically. Using a process dissociation procedure, we show in the present study that regional and foreign languages weaken the contribution of the deontological view in similar ways. Furthermore, the effects of both languages were modulated by proficiency, so that less proficient bilinguals showed a stronger decrease of the deontological tendency. These similarities suggest that the effects induced by both languages stem from what these languages have in common. Both languages are not experienced in contexts critical in forging moral views (e.g., public institutions, media, schools). We propose that the effects of foreign and regional languages stem from the lack of experience in such contexts.

## Introduction

The judgments we formulate about our own actions, or those committed by others or the society at large are often determined by moral views informing us of what is right and wrong, good or bad. Psychological research has shown that judgments driven by common morality can change depending on the circumstances (Bartels, Bauman, Cushman, Pizarro, & McGraw, 2015; Malle, 2021; Petrinovich & O’Neill, 1996). Evidence illustrating this variability was accrued, for example, with dilemmas created by philosophers as argumentative tools and later employed by psychologists as experimental devices. The trolley dilemma and the footbridge dilemma famously pose the same quandary: Is it morally acceptable to sacrifice the life of an individual to save the lives of others? (Foot, 1967; Thomson, 1985). The sacrifice occurs by turning a signal switch in the trolley dilemma, by pushing a bystander in the footbridge dilemma. Respondents are typically willing to turn the signal switch but quite resistant to push the bystander (Greene, Sommerville, Nystrom, Darley, & Cohen, 2001; Hauser, Cushman, Young, Kang-Xing Jin, & Mikhail, 2007; Petrinovich, O’Neill, & Jorgensen, 1993; Sheskin & Baumard, 2016). Decisions vary here depending on the circumstances – for example, whether the sacrifice requires physical contact or a device eliminating any close interactions (Cushman, Young, & Hauser, 2006; Greene, Cushman, Stewart, Lowenberg, Nystrom, & Cohen, 2009). Responses to these dilemmas further reveal that the weight of the moral views determining the decisions may also vary contextually. People subscribing to the moral imperative “do not kill” would do so only in some circumstances, the same for people whose morality is rooted in cost-benefit considerations. Language should also be listed among the contextual variables affecting moral judgments. As shown by a number of experiments, people may overcome their resistance to push the bystander when the footbridge dilemma is presented in a foreign language they had typically learned in school and have used less regularly and proficiently relative to their primary, native language (Costa, Foucart, Hayakawa, Aparici, Apesteguia, Heafner, & Keysar, 2014; for reviews, see Circi, Gatti, Russo, & Vecchi, 2021; Del Maschio, Crespi, Peressotti, Abutalebi, & Sulpizio, 2022; Stankovic, Biedermann, & Hamamura, 2022).

How do foreign languages affect moral views? To answer questions related to morality, psychologists have often turned to the distinction between deontology and utilitarianism that has permeated the philosophical debate (Conway & Gawronski, 2013; Greene et al., 2001; Kahane, Everett, Earp, Caviola, Faber, Crockett, & Savulescu, 2018). According to deontology, whether an action is right or wrong depends on whether it conforms to certain norms or values, regardless of the consequences. By contrast, as an instance of consequentialism, utilitarianism requires balancing costs and benefits. Utilitarianism specifically requires that actions aim to promote the greatest good for the greatest number. Deontology and utilitarianism often proscribe opposing actions, as in the case of the footbridge dilemma. Deontology forbids sacrificing the bystander, an action violating the norm “do not kill.” In contrast, utilitarianism prescribes harm if beneficial, as in this case in which killing one individual represents the action benefitting the largest number of people. Foreign languages would increase the acceptability of the sacrificial choice either by making the deontological view less appealing or by promoting the instrumental harm the utilitarian perspective demands. Hayakawa, Tannenbaum, Costa, Corey, and Keysar (2017) tested these alternatives using a process dissociation procedure devised by Conway and Gawronsky (2013) that assesses the contribution of deontological and utilitarian views in sacrifice-like dilemmas. Collectively, the results of six experiments revealed that foreign languages demoted the deontological view (for similar results see Białek, Paruzel-Czachura & Gawronsky, 2019; Muda, Niszczota, Białek, & Conway, 2018).

Miozzo, Navarrete, Ongis, Mello, Girotto, and Peressotti (2020) extended the investigation of the language effect on moral decisions to Italian regional languages. Sociolinguistically, these languages differ from foreign languages in several respects. They are acquired in infancy and spoken frequently and proficiently, in contrast to foreign languages that are typically learned at a later point in life and spoken less commonly and confidently. Italian regional languages are used among family, friends, neighbors, and acquaintances, and are almost exclusively oral (Maiden, 1995; Tuttle, 1997). Italian, the national language, is compulsory in civic institutions, schools, and churches, and represents the exclusive choice in media. The defining sociolinguistic features of Italian regional languages are shared with languages used by millions around the world (Kirby, Gray, Greenhill, Jordan, Gomes-Ng, Bibiko, et al., 2020). The reason for investigating Italian regional languages was not just to characterize the effect of a type of language so commonly used, but also the opportunity they offer to understand how language affects moral judgments. Italian regional languages (Venetian and Bergamasque) increased the rates of sacrificial choices in the footbridge dilemma (Miozzo et al., 2020), just as found with foreign languages. At face value, this similarity suggests that language effects are associated with features shared by these languages rather than features setting them apart. Foreign languages and Italian regional languages are both scarcely used in school, civic institutions, churches, and public media (Maiden, 1995; Tuttle, 1997), contexts providing experiences that forge people’s morality. If the language with which the moral judgments are framed facilitates the retrieval of values and norms associated with that language, then values and norms that are integral to common morality would be less available when foreign languages and Italian regional languages are used (Geipel, Hadjichristidis, & Surian, 2015; Miozzo et al., 2020). A prominent explanation attributes the effect of foreign languages to the weakness with which they elicit emotion (Harris, Ayçiçegi, & Gleason, 2003), which in turn makes emotion less able to promote certain judgments (e.g., the rejection of the sacrificial option in the footbridge dilemma) (Costa et al., 2014; Geipel et al., 2015; Kyriakou et al., 2022; Pavlenko, 2017). Italian regional languages do not trigger emotion to a lesser extent than the national language; nonetheless, like foreign languages, they increased the rate of sacrificial choices (Miozzo et al., 2020).

That foreign and Italian regional languages promote sacrificial choices does not imply that both languages have similar effects on moral judgments. While foreign languages appear to reduce the weight of deontological views, it is possible that Italian regional languages promote the utilitarian view. In this way, foreign and Italian regional languages would both increase the rate of sacrificial choices in moral dilemma, although for different reasons – foreign languages by affecting the deontological view, Italian regional languages by affecting the utilitarian view. Establishing whether foreign and Italian regional languages affect moral views in the same way is critical for explanations of the language effects, as it would rule out explanations like the one proposed by Miozzo et al. (2020) that assume that these two types of languages behave alike. It is presently unknown if Italian regional languages reduce the weight of deontological views or make utilitarian views more salient. This question was addressed in the present study in which we extended the process dissociation procedure used by the Hayakawa et al. (2017) to Venetian, a regional language spoken in the northeast part of Italy. To compare regional and foreign languages more closely, we also analyzed the effect of foreign languages. To this end, a second group of Italian-English bilinguals was included. In Italy, English is a foreign language usually acquired in school and used in daily activities rather sporadically, if at all, by those who learned it.

The process dissociation procedure devised by Conway and Gawronsky (2013) is based on responses to dilemmas that query whether it is morally acceptable to carry out specific actions. The procedure is designed to estimate the relative contribution of deontological and utilitarian perspectives in determining the judgments. Specifically, it provides two parameters describing the degree to which responses are driven by deontology (D) or utilitarianism (U). Both parameters increase as decisions rely on the perspectives they are associated with. Together, D and U allow researchers to gauge the strength of deontological and utilitarian perspectives. Measuring how D and U change across languages would inform us on whether language effects result from an increase or a decrease of the strength of each of these perspectives.

We focused in the present study on four findings that Hayakawa et al. (2017) reported with foreign languages, and our aim was to determine if they replicated with Italian regional languages. The first finding related to D. In all the six experiments conducted by Hayakawa et al. (2017), foreign languages induced lower D scores compared to native languages, thus revealing a reduced endorsement of deontology with foreign languages. The second finding concerned U. Foreign languages had weaker effects on U, a finding that is not in line with the hypothesis that foreign languages enhance sacrificial choices by strengthening the endorsement of the utilitarian view that promotes instrumental harm. However, lower U scores were found in only three of the six experiments. We examined if Italian regional languages had similarly contrasting effects on D and U – that is, if they lowered D with weaker effects, if any, on U. The third finding related to proficiency in the foreign language. The decrease of U scores observed with foreign languages was stronger among the less proficient participants, a finding Hayakawa et al. (2017) tentatively attributed to an increase in cognitive load experienced by participants with less familiarity with foreign languages. D scores, however, did not change as an effect of proficiency. We therefore examined the effect of proficiency in our study as well, although we departed slightly from Hayakawa et al. (2017) because in addition to examining self-rated proficiency, as they did, we assessed participants’ proficiency. Self-rated proficiency scores could be inaccurate and subject to contextual biases (Heilenman, 1990; MacIntyre, Noels, & Clément, 1997; Sitzmann, Ely, Brown, & Bauer, 2010), so that our change aimed to characterize the contribution of proficiency more precisely. The fourth finding related to incongruent dilemmas. As described in detail in the Methods section, incongruent dilemmas represent one type of dilemmas examined in the process dissociation procedure. These are the dilemmas traditionally investigated in the psychology research on morality; a typical example is represented by the footbridge dilemma. Hayakawa et al. (2017) did not find an effect of foreign languages on incongruent dilemmas. This finding is surprising if compared to the large body of experiments showing an effect of foreign languages on the footbridge dilemma (Circi et al., 2021; Del Maschio et al., 2022; Stankovic et al., 2022). As suggested by Hayakawa et al. (2017), the contrasting findings could stem from methodological differences, specifically that their participants responded to different kinds of dilemmas. This explanation is consistent with results from several studies showing that responses to inconsistent dilemmas changed when these dilemmas were presented with other types of dilemmas rather than alone (Bartels, 2008; Cushman et al., 2006; Lombrozo, 2009; Paharia, Kassam, Greene, & Bazerman, 2009; Petrinovich & O’Neill, 1996). The last finding examined in our study therefore related to incongruent dilemmas and we aimed to verify whether the lack of an effect found with foreign languages would be replicated with regional languages.

## Methods

### Materials and Procedures

#### a. Moral dilemmas

Two variants of the same dilemma were analyzed in the process dissociation procedure devised by Conway and Gawronsky (2013). Each variant described a similar scenario in which a harmful action would avert an undesirable outcome. In one scenario, for example, the harmful action was torture, which would be used to extoll information in order to prevent further crimes. The net benefit of the harmful action varied between the two variants of the dilemma. Incongruent dilemmas were designed to have the action causing a greater benefit than its harm. Torture of one person, for example, would avoid the explosion of bombs potentially injuring several people (see Table 1). Deontological and utilitarian views are here expected to promote opposing appraisals of the harmful actions. The benefit of harmful action was more trivial in congruent dilemmas – torture would avert the detonation of bombs spraying paint on nearby objects. Deontological and utilitarian views would converge here in rejecting the harmful action. We used the 10 pairs of congruent-incongruent dilemmas tested by Hayakawa et al. (2017) in Experiments 1 and 2, originally from Conway and Gawronski (2013). For Experiments 3–6, Hayakawa et al. (2017) tested another set of similar pairs, nevertheless they replicated the results of their Experiments 1 and 2.

**Table 1:** Example of congruent and incongruent variant of a dilemma (from Conway and Gawronsky, 2013).


INCONGRUENT	CONGRUENT

You are a police officer, and have recently caught a criminal you have been hunting for some time. He is allegedly responsible for rigging a series of explosive devices: some that have already gone off and some that have yet to detonate. He places explosives outside city cafes and sets them to go off at a time when people are drinking coffee on the patios. In this manner, he has injured many people and might injure many more. Now that the criminal is in custody, you want to know where the unexploded bombs are so you can defuse them. He refuses to talk, so you decide to use “aggressive interrogation techniques” like holding his head under water and beating him. Is it appropriate for you to use “aggressive interrogation techniques” in order to find and defuse the unexploded bombs?	You are a police officer, and have recently caught a criminal you have been hunting for some time. He is allegedly responsible for rigging a series of explosive devices: some that have already gone off and some that have yet to detonate. He places explosives outside city cafes and sets them to go off at a time when no one is around. His explosives are inside paint cans so that they spray nearby objects with paint. In this manner, he has sprayed many cafes with paint and might spray many more. Now that the criminal is in custody, you want to know where the unexploded bombs are so you can defuse them. He refuses to talk, so you decide to use “aggressive interrogation techniques” like holding his head under water and beating him. Is it appropriate for you to use “aggressive interrogation techniques” in order to find and defuse the unexploded bombs?


Because regional languages tend to have smaller vocabularies relative to national languages, the dilemmas from Conway and Gawronski (2013) were first translated from English into Venetian. The Venetian version was then translated into Italian. Next, the Italian version was translated into English. To verify the equivalence of the translations, bilinguals proficient in English back-translated the English version into Italian and Venetian.

Dilemmas were presented in different formats in Venetian (spoken) and English (written), to accommodate to variations in the acquisition and use of these languages. An almost exclusively oral language, Venetian only allowed a verbal presentation. In Italy, the teaching of foreign languages relies more on texts than spoken communication (Costa & Albergaria-Almeida, 2015; Faez, 2011), with the effect that written comprehension is better than spoken comprehension, as revealed by standardized national surveys (INVALSI, 2019). To maximize comprehension in the foreign language, we showed the dilemmas in written English. Previous studies of the language effect did not find reliable differences between spoken and written presentations. While Brouwer (2019) reported a language effect only when participants listened to moral dilemmas, Muda, Pieńkosz, Francis, and Białek (2020) failed to replicate this result when they re-analyzed the responses from Brouwer (2019) and repeated Brouwer’s (2019) experiment. Furthermore, in a more recent study (Brouwer, 2020), the language effect was not found to vary between modalities.

To keep the same format within each bilingual group, in Italian, too, we presented the dilemmas in a spoken or written format. In the spoken condition, dilemmas were recorded by four different speakers (two native speakers of Italian without knowledge of Venetian, and two very proficient Italian-Venetian bilinguals). For each language we used a male and a female voice. Dilemmas were presented in a spoken format to all Italian-Venetian bilinguals and in a written format to all Italian-English bilinguals. The materials are accessible on the Open Science Platform through the following link: https://osf.io/5y9k4/.

At the beginning of the experiment, participants in the Italian-Venetian group were informed to find a silent place or to wear headphones while participating in the experiment. Dilemmas were shown individually. To listen to each dilemma, participants in the Italian-Venetian group clicked on the play key (a rightward arrow) that appeared on the screen; they could listen to each dilemma multiple times by re-clicking on the play key. After each dilemma, participants were asked to indicate whether the described action was appropriate. The same question was asked in Conway and Gawronsky (2013) and in Experiments 1 and 2 of Hayakawa et al. (2017). Slightly different questions were used by Hayakawa et al. (2017) in Experiments 3–6, a variation that did not change the language effect. Two options (“Yes” and “No” and their translations in Italian and Venetian) appeared written on the screen. Participants chose their options by mouse clicking or screen touching, depending on the device they used for the task. To ensure that participants tested in English comprehended the dilemmas, they were instructed to select the option “I do not understand the English language” for any dilemma they failed to fully comprehend, an option selected for only 0.27% of the dilemmas (responses to these dilemmas were excluded from analyses). Additionally, at the end of the presentation of the dilemmas, they were asked to rate their comprehension of the dilemmas using a 10-point scale. Responses of participants who scored below 5 were considered unreliable and excluded from analyses. Participants tested in Venetian were not asked to rate their comprehension because, as described below, only highly proficient Venetian speakers were included in this group. As in Conway and Gawronsky (2013), the dilemmas were presented in a fixed pseudo-random order, so that the congruent and the incongruent version of a given dilemma were separated by the presentation of at least two other dilemmas.

#### b. Assessment of language proficiency and use

We assessed proficiency in English and Venetian through both testing and self-evaluation. Hayakawa et al. (2017) only used self-evaluation. Testing was added to achieve a more accurate assessment. English proficiency was evaluated with 10 questions selected from The Cambridge English Test assessing the reading competence of English as a second language (https://www.cambridgeenglish.org/test-your-english/general-english/). Questions of The Cambridge English Test examined different proficiency levels – from A1 (basic) to C1 (advanced). To vary the degree of difficulty, we included two questions from each proficiency level. The questions selected for our test examined both lexical knowledge and grammatical competence. Participants read one question at the time and selected the correct option among three or four alternatives. Proficiency was assessed in Venetian through an 8-item forced-choice grammaticality test from Miozzo et al. (2020) in which participants selected the correct response between two auditorily presented alternatives. This test was also useful to identify those participants who were not speakers of Venetian but, because of living in the Veneto region, they become somewhat familiar with it and used the partial resemblance with Italian to guess the meaning of the items. Participants with low scores on this test (below 5) were excluded from the analyses.

We administered the Language Use Questionnaire (Scaltritti, Peressotti, & Miozzo, 2017) to collect (a) information about age of acquisition and (b) self-reported proficiency and use for English or Venetian. Participants reported whether they learned the language before the age of 5 years. They also reported their proficiency in comprehension and production of English (or Venetian) on a 10-point scale, where 1 corresponded to “no competence” and 10 to “perfect competence.” A self-rated proficiency score was created by averaging comprehension and production scores. Participants were asked to estimate the percentage of time in which they used English (or Venetian) in different contexts (within family, with friends, in conversations with people for the same city/town, and at work) and for reading and watching films or videos. This last question was not included for Venetian, given that this language is almost completely oral and not used in the media.

#### c. Task administration

The study was conducted online. During the recruitment, participants were informed about the nature and duration of the task. They were then randomly assigned to one language (Italian or Venetian; Italian or English), and the dilemmas were introduced and presented in that language. Next, they completed the tasks assessing the acquisition, proficiency, and use of the language (Venetian or English) tested in their group. A final written question asked whether they consented to use their data for research. While dilemmas were presented in different languages, only Italian was used for the other sections of the study with all participants. Stimuli presentation and response recording were operated through Qualtrics (https://www.qualtrics.com). The research protocol was approved by the Ethical Committee for Psychological Research of the University of Padova (Protocol n. 3701).

### Participants

The study was advertised through social media and addressed Italian native speakers with knowledge of either English or Venetian. Separate contact chains were created for recruiting the two bilingual groups, one tested in Italian and English, the other in Italian and Venetian. The sample size appropriate for our experiment was determined using the a-priori sample size calculator for linear multiple regression available in G*Power (Version 3.1.9.6; Faul et al., 2007; 2009). Parameters were as follow: effect size = 0.03, number of tested predictors = 9, alpha level = 0.5, and power = 0.8. The recommended minimum sample size was 531 participants.

We excluded from analyses participants who did not complete the dilemmas and/or the other tasks assessing language proficiency and use, or did not match the inclusion criteria. Seven participants were excluded in the Italian-Venetian bilingual group because of scores below 5 in the Venetian proficiency test. Two participants were excluded in the Italian-English bilingual because of poor comprehension scores (<5/10). Data were analyzed from 298 Italian-Venetian bilinguals (152 tested in Italian, 146 in Venetian), and 310 Italian-English bilinguals (175 tested in Italian, 135 in English). Demographic and sociolinguistic variables were comparable between participants tested in Venetian and Italian or in English and Italian (see Table 2).

**Table 2:** Gender (% females), mean age (N years), percentage of participants who acquired English/Venetian before age 5, mean self-estimated proficiency in English/Venetian, and mean percentage of use of English/Venetian, across groups and languages. Standard deviations are reported in parenthesis.


GROUP	N	% FEMALES	AGE	BEFORE 5 YEARS	PROFICIENCY^a^	USE^b^

*Italian-English*						

Tested in Italian	175	77.1%	28.5 (10.41)	5.7%	7.5 (1.60)	30.9% (21.92)

Tested in English	135	73.3%	27.9 (9.93)	3.0%	7.8 (1.10)	34.3% (19.97)

*Italian-Venetian*						

Tested in Italian	152	67.8%	35.7 (15.29)	75.7%	7.7 (2.18)	35.6% (26.31)

Tested in Venetian	146	59.6%	33.2 (13.54)		7.9 (2.00)	37.8% (26.51)


^a^ Self-rated proficiency rated on a 10-point scale (1 = no competence; 10 = perfect competence). ^b^ Time (%) during which participants reported using the language, averaged across contexts.

**Figure 1:**
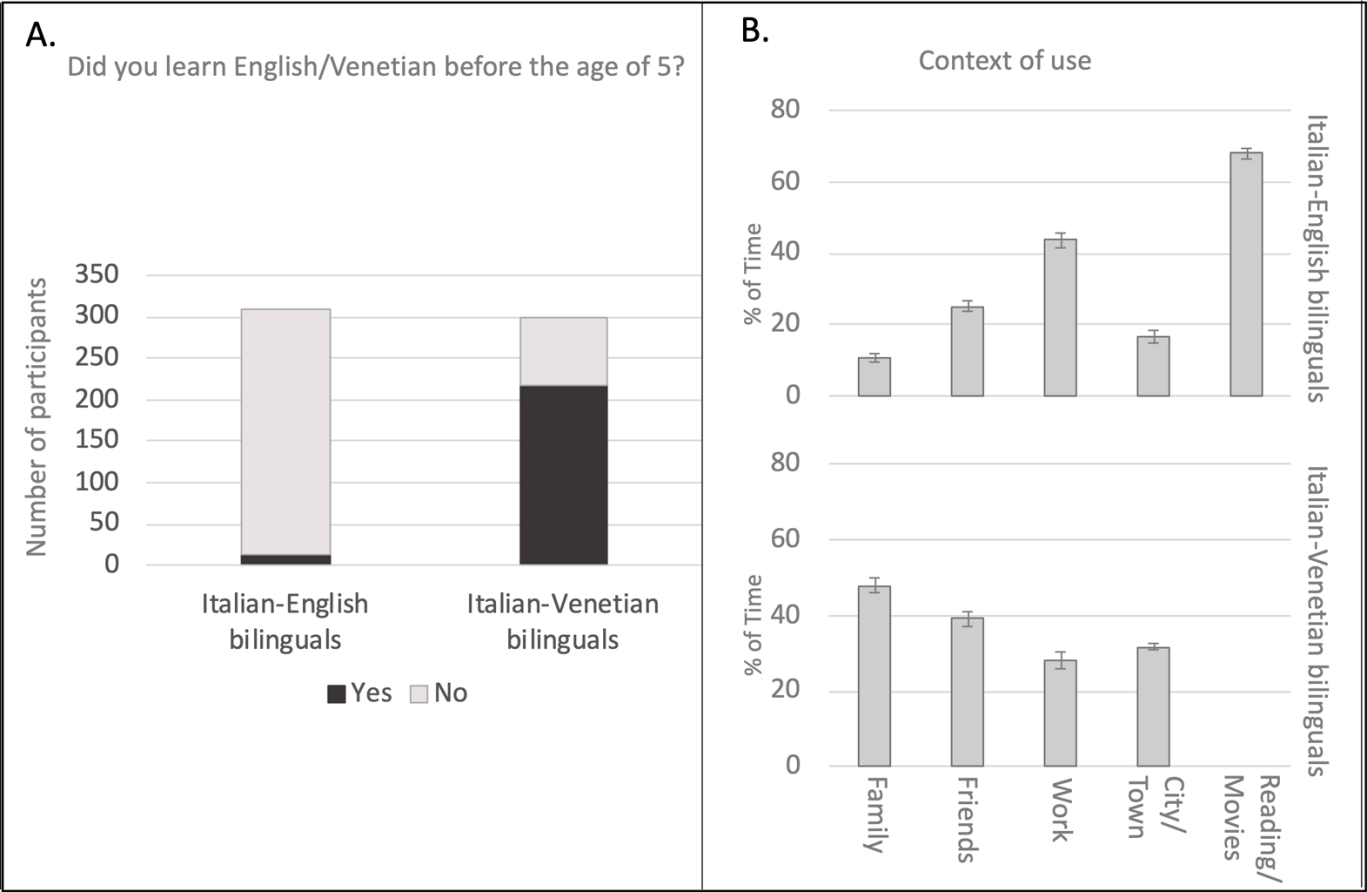
Panel A. Number of participants who learned English (or Venetian) before the age of 5 in each bilingual group. Panel B. Mean percentage of time during which Italian-English bilinguals reported using English (upper chart) and Italian-Venetian bilinguals using Venetian (lower chart) in different everyday-life contexts. The time spent reading and watching movies and videos was not estimated for Venetian because this language is essentially oral and not used in the media. Bars indicate standard error.

We expected the two bilingual groups to differ demographically and linguistically. Italian-Venetian bilinguals were older (mean (sd) = 34.5 (14.5) vs. 28.2 (18.2); t(606) = 6.165, p < .0001), and males were more represented within this group (36.2% vs. 26.9%, χ^2^ = 16.555, p < .0001). These differences aligned with census data, which showed that Italian regional languages are more used among older, male speakers (ISTAT, 2014). On the other hand, acquisition before the age of 5 was more common for Venetian than English (73.2% vs. 4.5% of participants; χ^2^ = 178.83, p < .0001; see Figure 1, Panel A). Proficiency was also greater in Venetian. Accuracy in the proficiency test was higher in Venetian than English (93.6% vs. 70.9%; t = 17.37, p < .0001), a difference, however, that we did not find with the self-rated proficiency scores (7.65 vs. 7.88; t = 1.182, p = .238). Further differences concerned the amount of time that participants reported using the two languages in various contexts (Figure 1, Panel B). Venetian is spoken especially in informal contexts, like among the family (47%) or friends (39%), unlike English that is used particularly at work (43.5%) or for reading and media consumption (67.8%). The large amount of time participants reported reading and watching films and videos in English is surprising if compared to how less commonly English was experienced in the other contexts. This could reflect the composition of our group – many of our participants were university students who had to read English textbooks. Furthermore, our participants were probably frequently exposed to English while surfing the internet or watching films subtitled in Italian. An advanced knowledge of English is probably not required in most of these cases, so that the time spent reading and watching films and videos in English should not be taken as an index of skillful reading and comprehension.

### Statistical Analyses

A first type of analyses concerned the dissociation procedure devised by Conway and Gawronsky (2013) to measure the strength of deontological and utilitarian perspectives within a participant. We used their equations to determine the scores for the utilitarian perspective (U) and deontological perspective (D). Both scores were based on the probabilities of rejecting harm in congruent and incongruent dilemmas. Specifically, the equations for U and D were:

U = p(unacceptable | congruent) – p(unacceptable | incongruent)

D = p(unacceptable | incongruent)/(1–U)

D and U scores were obtained for each participant included in the study. Following Hayakawa et al. (2017), we analyzed D and U scores separately. A Standard Least Square regression model was used to analyze D and U scores, respectively. Consistently with the purposes of our investigation, the model aimed to determine whether D and U scores varied between languages and, critically, whether the language effect varied between bilingual groups, in addition to establish the effect of language proficiency. To this end, the model we used included three predictors – i.e., Group (Italian-Venetian bilinguals vs. Italian-English bilinguals), Language (Italian vs. English or Venetian), and Proficiency (% correct responses in the language’s proficiency test) – as well as the following interactions: Group × Language, Group × Proficiency, Proficiency × Language, and Group × Language × Proficiency. Responses to moral dilemmas were found to vary as an effect of age and gender (Baez, Flichtentrei, Prats, Mastandueno, García, Cetkovich, & Ibáñez, 2017; Friesdorf, Conway & Gawronski, 2015; Conway, Goldstein-Greenwood, Polacek, & Greene, 2018; Rosen, Brand & Kalbe, 2016). In consideration of their effects, the factors Age and Gender were added as predictors to the model (with the exclusion of 13 participants who elected not to choose a gender).

A second type of analyses focused on the incongruent dilemmas, and were identical to the analyses traditionally conducted on the moral dilemmas. They were based on the rate of utilitarian responses – i.e., responses in which harmful actions were judged as appropriate. The Standard Least Square regression model created for these analyses aimed to determine whether responses varied in English (or Venetian) with respect to Italian, and whether English and Venetian had comparable effects. To this end, Group (Italian-Venetian bilinguals vs. Italian-English bilinguals), Language (Italian vs. English or Venetian), and the Language × Group interaction were entered as predictors. Because age and gender demonstrated to affect responses to moral dilemmas (Baez et al., 2017; Friesdorf et al., 2015; Conway et al., 2018; Rosen et al., 2016), both variables were added to the model. The full output of statistical analyses is available on the Open Science Platform through the following link: https://osf.io/5y9k4/.

All analyses were conducted with the JMP Pro16.2.0 program.

## Results

Congruent dilemmas were expected to be vastly rejected, as they were designed to be conflicting with both deontological and utilitarian views. The congruent abortion dilemma, which queried about an abortion performed to avoid a potential socioeconomical burden, defied this expectation, as most participants (78.5%) judged the abortion to be the appropriate choice. All other compatible dilemmas had lower acceptance rates, ranging between 1.1% and 39.8%. We therefore excluded from analyses both variants of the abortion dilemma. Among the remaining dilemmas, harmful actions were overall judged more appropriate on the incongruent dilemmas compared to the congruent dilemmas (means: 48.8% vs. 15.9%; t(1214) = 34.877, p < .0001).

As a validity check, we examined if D and U scores were uncorrelated, as predicted by the theory underlying the process dissociation procedure (Jacoby, 1991) and previously found (Conway & Gawronsky, 2013; Friesdorf et al., 2015; Hayakawa et al., 2017). D and U scores were weakly correlated (r = 0.11). As a further validity check, we examined how the utilitarian responses given to incongruent dilemmas correlated with D and U scores. In line with previous findings (Conway & Gawronsky, 2013; Friesdorf et al., 2015; Hayakawa et al., 2017), they correlated negatively with D scores (r = –0.64) but positively with U scores (r = 0.66).

### a. Parameter D

**Figure 2:**
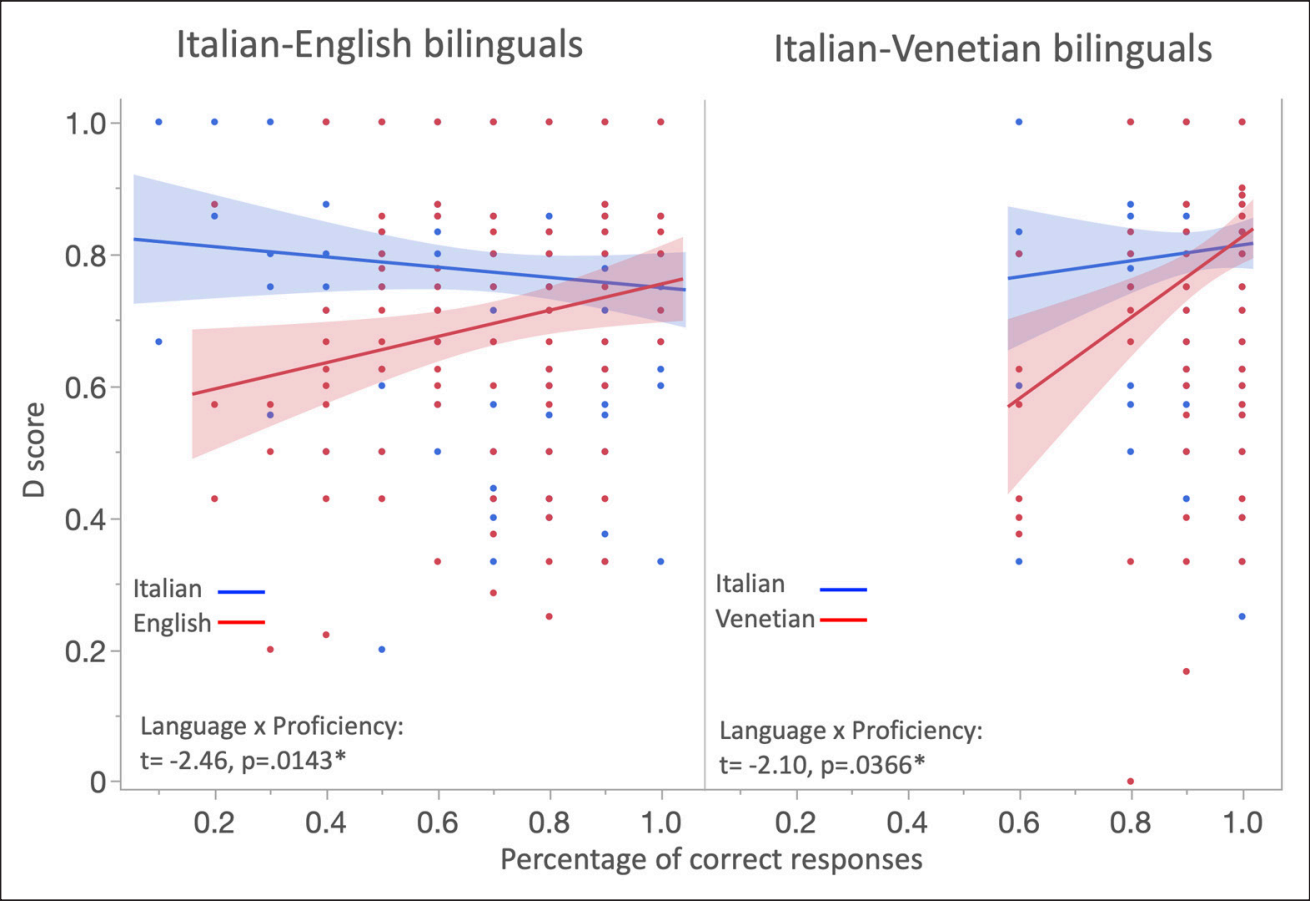
D scores of Italian-English bilinguals and Italian-Venetian bilinguals as a function of the percentage of correct responses in tests assessing proficiency in English (left) and Venetian (right). Even when analyzed separately, both bilingual groups showed significant Language × Proficiency interactions (t and p values are reported in the bottom left portion of each panel).

Analyses revealed a significant effect of Language (t = 2.97, p = 0.0081, η_p_^2^ = 0.012), reflecting the lower D scores that, compared to Italian, were found in English (estimated means (standard errors): 0.690 (0.020) vs. 0.739 (0.018)) and Venetian (estimated means, (standard errors): 0.701 (0.025) vs. 0.766 (0.024)). Noticeably, the lack of a significant Group × Language interaction (t = -.38, p = .403) further indicated that English and Venetian lowered D scores to comparable degrees. Together, these results indicated that English and Venetian weakened the contribution of the deontological perspective. Analyses also revealed a significant effect of Proficiency (t = 2.97; p = 0.003; η_p_^2^ = 0.015), which was qualified by the interaction of Proficiency with both Language (t = –2.91; p = 0.0037; η_p_^2^ = 0.014) and Group (t = –2.68; p = 0.0075; η_p_^2^ = 0.012). As illustrated in Figure 2, the Proficiency × Language interaction emerged because the effect of proficiency appeared in English and Venetian but not Italian. The interaction among Group, Language and Proficiency was not significant, indicating that the proficiency influenced the language effect in a similar manner across both groups of bilinguals. To support this conclusion, we conducted separate analyses for the two groups of bilinguals, using a regression model with Language, Proficiency, and Language × Proficiency interaction as predictors. Both models revealed a significant Language × Proficiency interaction (Italian-English bilinguals: t = –2.47, p = .014, η_p_^2^ = 0.016; Italian-Venetian bilinguals: t = –2.10, p = .036, η_p_^2^ = 0.021; see Figure 2). We can therefore conclude that in both English and Venetian, D scores increased with proficiency; in addition, as suggested by the Proficiency × Group interaction, the increase was more marked in Venetian than English. Finally, significant effects of Age (t = 4.22; p < 0.0001; η_p_^2^ = 0.029) and Gender (t = 5.31; p < 0.0001; η_p_^2^ = 0.046) were observed. D scores increased with age and were higher for female than male participants (estimated means (standard error): 0.772 (0.012) vs. 0.676 (0.017)). Both results replicated prior findings (Baez et al., 2017; Conway et al., 2018; Friesdorf et al., 2015; Muda et al., 2018; Rosen et al., 2016).

### Parameter U

Analyses only revealed a significant effect of Gender (t = –3.32; p = 0.001; η_p_^2^ = 0.018). Consistent with prior findings (Baez et al., 2017; Friesdorf et al., 2015; Conway et al., 2018; Rosen et al., 2016), U scores were higher for male than female participants (estimated means: 0.376 vs. 0.321). Nor the effect of language (t = 1.49; p = .137), nor the interaction Language × Proficiency (t = –1.19; p = .235) were significant. Mean U scores (and standard errors) were 0.365 (0.016) and 0.337 (0.018) for Italian and English, respectively, and 0.361 (0.022) and 0.331 (0.023) for Italian and Venetian, respectively.

### Responses to incongruent dilemmas

Analyses showed an effect of Group (t = 2.80; p = 0.0052; η_p_^2^ = 0.013), explained by the higher rates with which harmful actions were judged appropriate by Italian-Venetian bilinguals compared to Italian-English bilinguals (estimated means: 4.8 vs. 4.4).

**Figure 3:**
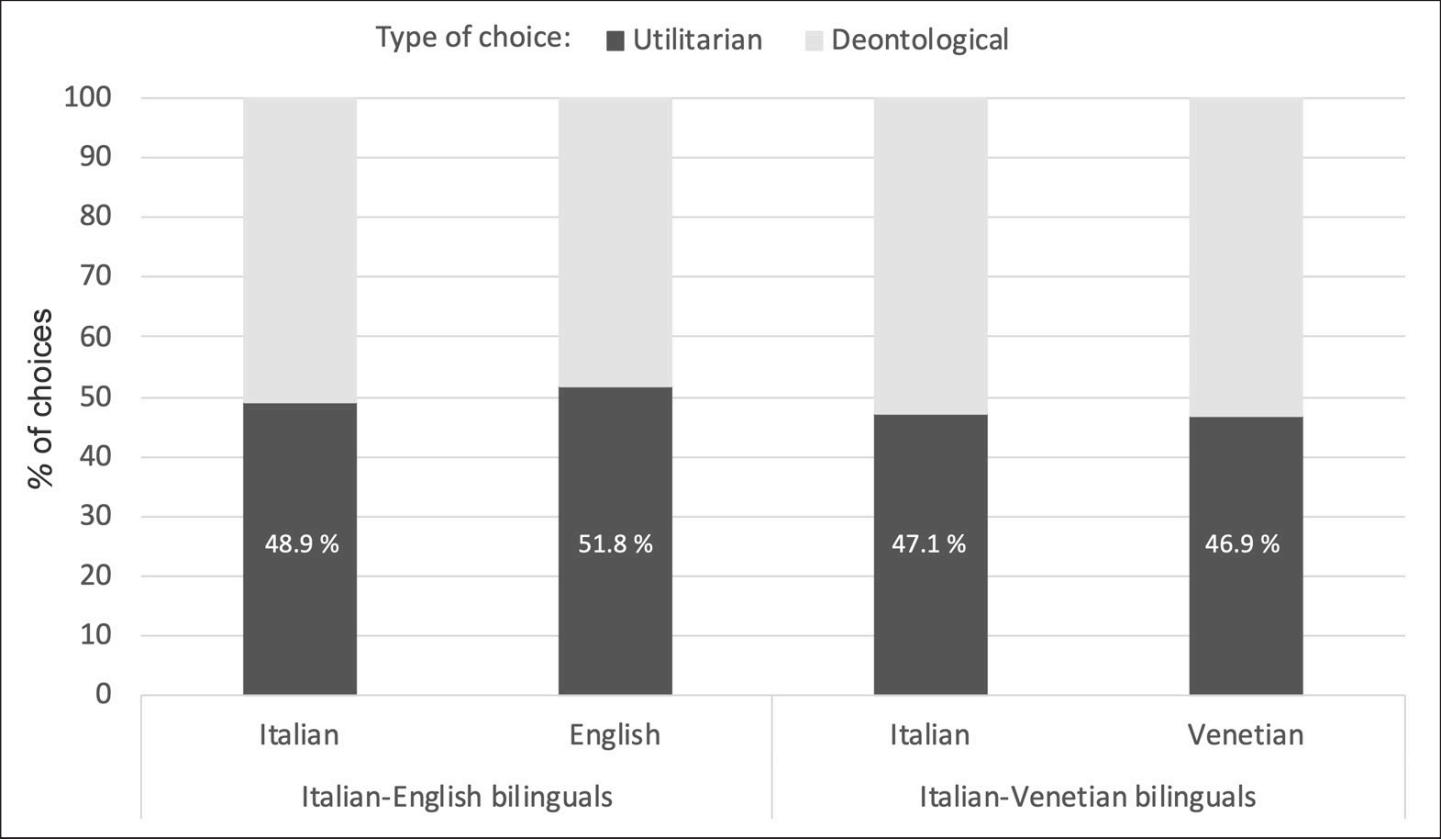
Percentage of sacrificial responses in incongruent dilemmas as a function of group (Italian-English bilinguals and Italian-Venetian bilinguals) and language comparison (Italian vs. English and Italian vs. Venetian). Percentage of dilemmas in which participants accepted the sacrifice (black bar) relative to those in which they rejected it (grey bar).

Notably, neither Language nor the Group × Language interaction were significant, suggesting that judgments did not vary as a function of language (Figure 3). In line with previous findings (Baez et al., 2017; Conway et al., 2018; Friesdorf et al., 2015; Rosen et al., 2016), Age and Gender were both significant. Harmful actions were judged more favorably by males than females (5.1 vs. 4.1; t = –6.36, p < .0001) and as age increased (t = –3.40; p = 0.001).

## General Discussion

English, the foreign language we tested, lowered the D scores but had null effects on the U scores, thus reducing the weight of the deontological inclination. Furthermore, English did not significantly affect decisions in incongruent dilemmas pitting the deontological against the utilitarian decisions. The same results with D scores and incongruent dilemmas were found in experiments carried out by Hayakawa et al. (2017), Muda et al. (2018) and Bialek et al. (2019), so that our replication confirmed their robustness. The novelty of our study is represented by its results with the Italian regional language. These results, too, fully replicated those reported by Hayakawa et al. (2017) with foreign languages. Furthermore, in both foreign and regional languages, the decrease in deontological responding was more marked for less proficient bilinguals.

A weaking of the endorsement of deontology can be explained in at least two ways. Under one explanation, information favoring deontological choices could be less available. This could happen with foreign and regional languages because of their limited use in some of the social contexts instrumental in shaping common morality (Geipel et al., 2015; Miozzo et al., 2020). To the extent that this explanation anticipates that foreign and regional languages affect moral views in similar ways, the convergence between foreign and regional languages shown by our results provide some support to it. The other explanation relates to the hypothesis that emotion promotes deontological choices (Costa et al., 2014; Pavlenko, 2017). Languages triggering weak emotion responses would make deontological choices less likely. This explanation applies to foreign languages that have been shown to induce a weak emotion response (Harris et al., 2003), not so much to regional languages that do not trigger an equally weak emotion response (Miozzo et al. 2020). The similarities between the effects of foreign and regional languages found here and in Miozzo et al. (2020) add to other results that did not align with the hypothesis linking the language effect to emotion (Geipel et al., 2015; Hadjichristidis, Geipel, & Savadori, 2015). While all these results do not exclude that emotion is involved in the language effect, they nevertheless suggest that emotion is unlikely to be the only source of the effect.

Conway and Gawronsky (2013) framed the process dissociation procedure within a dual-process account proposing that deontological judgments are driven by emotional processes, while utilitarian judgments are under the control of cognitive processes. This type of dual-process account has received empirical support from studies showing that choices became more deontological or utilitarian under the effect of experimental manipulations affecting emotion or cognitive processes (Amit & Greene, 2012; Bartels, 2008; Choe & Min, 2011; Moore, Clark, & Kane, 2008; Small & Loewenstein, 2003; Suter & Hertwig, 2011; Trémolière, De Neys, & Bonnefon, 2012). Further evidence was accrued with neuroimaging and lesion analyses by examining brain responses and lesions in areas associated with emotion and cognitive processes, respectively (Ciaramelli, Muccioli, Làdavas, & Di Pellegrino, 2007; Cushman, Murray, Gordon-McKeon, Wharton, & Greene, 2012; Greene et al. 2001; Greene, Nystrom, Engell, Darley, & Cohen, 2004; Koenigs, Young, Adolphs, Tranel, Cushman, Hauser, & Damasio, 2007). This dual-process account of morality, however, has been questioned on empirical as well as theoretical grounds (Bartels et al., 2015; Kahane et al., 2018; Kvaran & Sanfey, 2010; McGuire, Langdon, Coltheart, & Mackenzie, 2009; Moll, Zahn, de Oliveira-Souza, Krueger, & Grafman, 2005; Sheskin & Baumard, 2016). For example, alcohol intoxication, a condition impairing higher-order executive functioning, was found to increase utilitarian preferences (Duke, & Bègue, 2015). Similarly, time-pressure, which interferes with effortful reasoning, increased utilitarian responses (Rosas & Aguilar-Pardo, 2019). Furthermore, people reporting low levels of affective concerns – as established by higher scores on measures of psychopathy, machiavellianism, life meaninglessness or egoism – demonstrated greater support for universal, unconditioned care (Bartels & Pizarro, 2011; Gleichgerrcht & Young, 2013; Kahane, Everett, Earp, Farias, & Savulescu, 2015). While these results question the account within which Conway and Gawronsky (2013) framed the process dissociation procedure, they do not undermine the use of the procedure for the purpose of investigating deontology and utilitarianism. Other dual-process accounts have been proposed to explain the emergence of deontological and utilitarian judgments, including affect-backed rules (Nichols & Mallon 2006), protected values as affect-backed constraints (Bartels, 2008), and Cushman’s (2013) model based on reinforcement learning. They could represent adequate alternatives to describe deontological and utilitarian preferences. We should also note that our use of the process dissociation procedure was empirically motivated – our goal was to examine the replicability in Italian regional languages of effects found in foreign languages – and did not require we subscribe to any specific account of the process dissociation procedure.

The participants of our experiment read the moral dilemmas when tested in English but listen to them when tested in Venetian. This difference was dictated by the distinct use of each language. Venetian, a spoken language, could only be tested orally, whereas a greater familiarity, in Italy, with reading than listening English (INVALSI, 2019) determined we asked our participants to read the moral dilemmas when tested in English. To the extent that our findings showed that English and Venetian induced similar effects on the responses to moral dilemmas, the difference in presentation format does not seem to be critical. Additionally, when considering only the Italian condition, our design allowed us to directly test the effect of modality. Both bilingual groups responded in Italian, however the dilemmas were presented in a written form to Italian-English bilinguals and orally to Italian-Venetian bilinguals. No modality effect was found for D (t = –1.01) or U (t = –0.04) parameters (refer to Supplementary materials for a detailed description of these analyses). Whether presentation format could modulate language effects was investigated with foreign languages, but results were inconsistent. Brouwer (2019) reported a language effect when participants listened to the moral dilemmas but not when they read the moral dilemmas, a finding that Muda et al. (2020) failed to replicate when they re-analyzed the responses from Brouwer (2019) and that was not reproduced in two more recent experiments (Brouwer, 2020; Muda et al., 2020). The results we obtained in English confirmed that foreign languages affect responses to moral dilemmas presented in reading and that their effects are not confined to a spoken presentation.

We measured language proficiency in two ways – objectively, by testing the knowledge of lexical and grammatical features, and by means of self-reporting. Only the objective proficiency measure was found to modulate the reduced endorsement of deontology observed with foreign and Italian regional languages (Figure 2). The lack of an effect with self-reported proficiency probably reflected the relative inaccuracy of this measure (Heilenman, 1990; MacIntyre et al., 1997; Sitzmann et al., 2010). Our data provided a hint of its imprecision. Italian-English bilinguals estimated their proficiency in English higher when they responded to the dilemmas in English rather than Italian (t(308) = 2.198, p = .0287; mean ratings = 7.85 vs. 7.50). By showing that a brief exposure to these languages changed their scores, these data expose the challenge that participants faced to provide an accurate assessment of their language skills. Our results with self-rated proficiency scores are the only findings differing from those reported by Hayakawa et al. (2017). While in their experiments U scores varied as an effect of self-rated proficiency, this variable had no effects in our study either with the foreign language or the regional language. The effects of proficiency we observed in both languages appeared instead with objective measures of proficiency. Even if the reasons of these discrepancies are unclear, the results from both studies concur in showing that proficiency could modulate the language effect, and in this respect is a variable researchers should consider controlling. It is also noteworthy that proficiency effects arose in languages differing from each other in terms of acquisition and use. These similarities make it more compelling that the same mechanisms underlie the effect of languages varying so widely.

As in previous studies (Bialek et al., 2019; Hayakawa et al., 2017; Muda et al., 2018), language effects were found with the process dissociation procedure, but they did not appear with the forced-choice (appropriate/inappropriate) judgments given to the actions described in incongruent dilemmas, even when a regional language was used. This discrepancy has clear methodological implications. The process dissociation procedure appears to be a rather sensitive method capable of revealing language effects not observable from forced-choice judgments to incongruent dilemmas. Under this procedure, incongruent dilemmas are presented together with congruent dilemmas, and the two types of dilemmas prompt different kinds of responses. This mix could in part be responsible for the lack of language effect with incongruent dilemmas, as suggested by previous findings showing that responses to incongruent dilemmas changed depending on whether these dilemmas were presented alone or with other types of dilemmas (Bartels, 2008; Cushman et al., 2006; Lombrozo, 2009; Paharia et al., 2009; Petrinovich & O’Neill, 1996). The inclusion of different types of dilemmas should be carefully considered when designing tasks involving incongruent dilemmas.

Languages are not born equal. Exploring their differences may provide researchers a tool for understanding language mechanisms. This is the approach we took in examining the effects of language on moral judgments. The results we reported here confirmed that languages differing substantially for acquisition and use appear to affect moral decisions in similar ways. This consistency constraints explanations of the language effects on moral judgments. The cause of these language effects should be searched among the features that these languages share, and comprehensive descriptions of these effects would emerge from explanations that can incorporate such similarities.

## Data Accessibility Statement

The data that support the findings of this study are openly available in the Open Science Platform at https://osf.io/5y9k4/.
